# Bromide of Ethyl—the New Anæsthetic

**Published:** 1880-03

**Authors:** 


### SELECTIONS.

### BROMIDE OF ETHYL—THE NEW ANÆSTHETIC.

BROMIDE of ethyl or hydrobromic ether is a transparent colorless liquid, heavier than water, (sp. gn. 1.423). Its taste is sweetish and pungent, and its odor ethereal and not unpleasant. It is sparingly soluble in water, but mixes in all proportions with alcohol and ether. It is not caustic nor even irritant when compared with chloroform. Its anæsthetic action has long been known, but Dr. Lawrence Turnbull of Philadelphia, was the first to experiment with this ether upon man.

He publishes reports of twenty-one cases, and the advantages which he claims for this agent are: The rapidity of its elimination from the system by the respiratory passages; the small quantity required, and the readiness with which anæsthesia is produced.

Dr. Levis, Surgeon to the Pennsylvania Hospital, and to Jefferson College Hospital, expresses himself favorably as to its use; he says, "I have now had sufficient experience upon which to at least base some very decided impressions, as to its value. Its vapor is quite unirritating to the respiratory passages when inhaled, and in this quality it has the advantage over both ether and chloroform. It seems to be entirely eliminated through the lungs, and in cases in which some secreting organs happen to be from disease incapacitated, it has in this regard a decided advantage as to safety over chloroform.

"Its principal physiological characteristics, which will concern the surgeon, are its rapidity of action and the quickness of recovery from its effects; as far as observed by me it does not influence the circulation, excepting to produce sometimes a slight increase in the rapidity of the heart's action and in arterial tension or pressure.

"The cerebral anæmia and the fatal syncope of cardiac depression, to which chloroform is liable, are dangers which do not appear to threaten in the anæsthesia of the bromide of ethyl; nausea and vomiting appeared to occur less frequently. The action is very rapid—complete anæsthesia is accomplished in one-third less time than is the case with chloroform; usually the pupils dilate during total insensibility; but soon contract if the administration is interrupted. The effect is transient, in a few seconds after removing the towel, the patient is able to talk intelligently, and in a minute or two can walk away without a stagger.

"The quantity consumed will depend upon the manner of using it. Dr. Levis' plan is to pour two drams of the ethyl bromide on a small napkin folded up to a space of about four inches, and then laid on another napkin folded so as to be large enough to cover the entire face of the patient.

"So powerful an agent as bromide of ethyl, should be used with caution—but the results of experience so far demonstrate that it is a safe anæsthetic agent, quicker in action, more transient in effect—in some respects preferable to ether or chloroform."

